# Patient satisfaction with breast reconstructionusing musculocutaneous flap from latissimus dorsiversus from rectus abdominis: a cross-sectional study

**DOI:** 10.1590/1516-3180.2018.032916112018

**Published:** 2018-12-13

**Authors:** Lilian Baldan Záccaro Augustinho, Miguel Sabino, Daniela Francescato Veiga, Luiz Eduardo Felipe Abla, Yara Juliano, Lydia Masako Ferreira

**Affiliations:** I MSc. Physiotherapist, Faculdade de Filosofia Ciências e Letras de Catanduva (FAFICA), Catanduva (SP), Brazil; II MD, PhD. Physician, Adjunct Professor and Coordinator of the Postgraduate Program on Translational Surgery, Universidade Federal de São Paulo (UNIFESP), São Paulo (SP), Brazil.; III MD, PhD. Physician and Professor, Postgraduate Program on Translational Surgery, Universidade Federal de São Paulo (UNİFESP), São Paulo; and Pro-Rector of Postgraduate Studies, Universidade do Vale do Sapucaí (UNIVÁS), Pouso Alegre (MG), Brazil.; IV MD, PhD. Physician and Director, Discipline of Plastic Surgery, Hospital Pérola Byington, São Paulo (SP), Brazil.; V PhD. Business Administrator and Full Professor, Discipline of Collective Health, School of Medicine, Universidade de Santo Amaro (UNISA), São Paulo (SP), Brazil.; VI MD, PhD. Physician and Titular Professor, Discipline of Plastic Surgery, Universidade Federal de São Paulo (UNİFESP), São Paulo (SP), Brazil.

**Keywords:** plastic, Mammaplasty, Breast neoplasms, Health

## Abstract

**BACKGROUND:**

Breast cancer is the second most frequent type of cancer worldwide and the most common type among women. The treatment for this condition has evolved over recent decades with therapeutic and technological advances. Breast reconstruction techniques using musculocutaneous flaps from the latissimus dorsi and rectus abdominis have aroused interest regarding patients’ quality of life. Our goal here was to compare patients’ satisfaction scores after they underwent breast reconstruction using musculocutaneous flaps from either the latissimus dorsi or the rectus abdominis.

**DESIGN AND SETTING:**

Primary, clinical, analytical, observational and cross-sectional study conducted in a federal university and a public hospital.

**METHODS:**

Demographic and clinical data were collected. The Mini-Mental State Examination was then applied, with testing for specificity and sensitivity. Lastly, a breast evaluation questionnaire was applied to evaluate breast satisfaction among 90 women, who were divided into three groups: mastectomy (control; n = 30); breast reconstruction using flap from the latissimus dorsi (n = 30); and reconstruction using flap from the rectus abdominis (n = 30).

**RESULTS:**

The groups were homogeneous regarding the main demographic data and the questionnaire responses (P < 0.05). Compared with the control group, the reconstruction groups showed significant improvement in satisfaction (P < 0.0002) after one year.

**CONCLUSION:**

Within our sample, women who underwent breast reconstruction with flaps from either the latissimus dorsi or the rectus abdominis had similar satisfaction scores.

## INTRODUCTION

Breast cancer is the second most frequent type of cancer in the world and the most common type among women. Overall, it accounts for 28% of new cases of cancer.^1^ In developed countries, the five-year survival rate is about 85%, whereas in developing countries, it remains between 50 and 60%.^1^ It has been estimated that there will be 59,700 new cases of breast cancer in Brazil in 2018, i.e. 56.33 cases per 100,000 women.^1^

Therapeutic and technological advances relating to breast cancer have allowed patients to attain greater life spans, and this has drawn attention to the quality of life of these women.^2^^,^^3^^,^^4^ Mastectomy is considered to be one of the most devastating types of cancer treatment from a psychological point of view, in that it affects the self-esteem, femininity and body image of these patients.^5^^,^^6^ However,breast reconstruction can be undertaken immediately after mastectomy or as a delayed procedure. Breast reconstruction procedures have positive impacts on all aspects of quality of life, including body image, particularly among younger women.^2^^,^^7^

There are several types of breast reconstruction. The autologous reconstruction methods that are most used are breast reconstruction using a transverse rectus abdominis musculocutaneous flap and breast reconstruction using a latissimus dorsi flap, which provide safety and satisfaction for patients and improve their quality of life.^2^^,^^8^^,^^9^^,^^10^

Anderson etal. developed a specific questionnaire for patients with breast diseases (named the Breast Evaluation Questionnaire) that has proven to be reliable and valid. It contains 55 questions and was developed to assess patients’ satisfaction with their breasts and their contentment with their general appearance and the appearance of their breasts. This questionnaire thus reveals changes in the quality of life of patients who have undergone breast surgery.^11^ This instrument has been translated and validated for use in the Portuguese language.^12^

There is a gap in the literature concerning comparison of the results from these two reconstruction techniques, in relation to thepatients’ quality of life. This is what motivated the present study.

## OBJECTIVE

Our objective was to compare patients’ satisfaction scores after they underwent breast reconstruction using musculocutaneous flaps from either the latissimus dorsi or the rectus abdominis.

## METHODS

### Design, setting and ethics

This was a primary, clinical, analytical, observational and cross-sectional study. It was conducted at a federal university and a public hospital.

It was approved by the Ethics Committee of the Federal University of São Paulo on October 26, 2012, under the approval number 131.769. Patients signed informed consent forms for their inclusion in the study.

### Participants

The patients were recruited from the breast surgery outpatient clinic of São Paulo Hospital (which is linked to the discipline of plastic surgery within the Federal University of São Paulo) and from the breast surgery outpatient clinic of Perola Byington Hospital. The patients in the control group were selected from the plastic surgery outpatient clinic and went through the same scheduling and data collection process.

The sample was formed by 90 women aged between 30 and 55years, who were divided into three homogeneous groups according to the surgical technique used: 30 women who had undergone mastectomy without reconstruction (control group); 30 women who had undergone immediate or delayed breast reconstruction using a musculocutaneous latissimus dorsi flap (LD group); and 30 women who had undergone immediate or delayed reconstruction using a transverse rectus abdominis musculocutaneous flap (TRAM group).

The following inclusion criteria were taken into account in selecting the patients:

- Age between 30 and 55 years;
- Mastectomy (control group) with immediate breast reconstruction or breast reconstruction that had been delayed for up to one year (LD and TRAM groups);
- Completed adjuvant treatment for at least six months; and
- Score greater than or equal to 18 in the Mini-Mental State Examination. This test forms a practical method for evaluating cognitive function and tracking states of dementia. Ithas different cutoff scores: 13 points for illiterate people; 18 points for individuals with one to seven years of schooling; and 26points for people with eight years or more of schooling. Scores greater than 18 refer to people who are literate and able to understand and answer questions.^13^

The following were exclusion criteria:

- Neoadjuvant chemotherapy or adjuvant radiotherapy;
- Breast disease occurring during the study;
- Illiteracy;
- Recurrences or metastases.

### Breast evaluation instrument (breast evaluation questionnaire)

The Breast Evaluation Questionnaire (BEQ) evaluates patients’ satisfaction with their breasts, regarding breast size, shape, firmness and overall appearance, along with the appearance of their breasts when wearing clothes or swimsuits and when naked. Moreover, this questionnaire enables assessment of the importance of breast appearance for the patient and for other people.^11^^,^^12^

It comprises 55 items for which the patient selects one answer. These are grouped into 11 sections with five items in each section. Each item can be scored from one to five, as follows: very dissatisfied (score = 1); slightly dissatisfied (score = 2); neither satisfied nor dissatisfied (score = 3); reasonably satisfied (score = 4); or very satisfied (score = 5). The total score for the questionnaire is obtained by summing the scores given for each item in each section.

The data from the questionnaire were analyzed after standardization as percentages, because the score which each item receives can vary and, consequently, change the total score.^11^

$$\frac{Score:\left(total\;score-lowest\;score\right)\times 100}{Possible\;variation}$$

### Data collection procedure

The interviews for data-gathering were scheduled to take place at the time of the patients’ return visits to the outpatient clinic (i.e. in the cases of the LD and TRAM groups). At these meetings, the patients were oriented and were invited to take part in the study. If they agreed, they would sign an informed consent form.

Initially, sociodemographic and clinical data were gathered. Following this, the Mini-Mental State Examination and then the Breast Evaluation Questionnaire (BEQ-55) were applied. All questionnaires were self-administered in a reserved room, immediately after the patient’s medical consultation.

### Statistical analysis

To analyze the results, the BioEstat 5.0 software was used.

The following tests were applied:

- Kruskal-Wallis variance analysis, to compare the three study groups concerning the quantitative variables. When differences between the groups were significant, the analysis was complemented with multiple comparison testing to determine which group(s) differed from the other(s).^13^
- Chi-square test, to study associations between the groups and the characteristics observed.^13^

In all tests, the rejection level for the null hypothesis was set at 5%.

## RESULTS

Table 1 shows the absolute and relative frequencies for the categorical demographic variables (marital status, skin color, schooling and occupation) that were obtained from the patients in each study group, and the comparisons between the groups (chi-square test).

**Table 1.** Distribution of the women who underwent breast reconstruction, according to sociodemographic characteristics

		Control	%	LD	%	TRAM	%	Total	%	Chi-square test: P
Marital status	With partner	17	56.7	22	73.3	13	43.3	52	57.8	0.0621
	Without partner	13	43.3	8	26.7	17	56.7	38	42.2	
Skin color	Caucasian	20	66.7	19	63.3	19	63.3	58	64.4	0.9527
	Non-Caucasian	10	33.3	11	36.7	11	36.7	32	35.6	
Occupation	Housewife	6	20	6	20	8	26.7	20	22.2	0.6717
	Working outside the home	24	80	24	80	22	73.3	70	77.8	
Schooling	Elementary school	5	16.7	4	13.3	5	16.7	14	15.6	0.8315
	High school	16	53.3	20	66.7	19	63.3	55	61.1	
	Higher education	9	30	6	20	6	20	21	23.3	
Total		30	100	30	100	30	100	90	100	

LD = reconstruction using musculocutaneous flap from the latissimus dorsi; TRAM = reconstruction using musculocutaneous flap from the rectus abdominis.

Table 2 presents the data relating to numerical sociodemographic variables (BMI and age), and the comparisons between the groups (Kruskal-Wallis test).

**Table 2.** Age and body mass index (BMI) among the women who underwent breast reconstruction

		Control	LD	TRAM	Kruskal-Wallis test: P
BMI (kg/m²)	Range	20.1-25.9	20.3-27.2	20.4-26.9	0.4066
	Median	24.1	23.7	23.4	
	Average	23.9	23.3	23.6	
Age (years)	Range	30.0-52.0	34.0-55.0	36.00-55.0	0.4907
	Median	47	47	48	
	Average	45.4	46.7	46.9	

LD = reconstruction using musculocutaneous flap from the latissimus dorsi; TRAM = reconstruction using musculocutaneous flap from the rectus abdominis.

The analysis on individuals’ data obtained from application of the BEQ in the control-mastectomy group, the group with breast reconstruction using a musculocutaneous flap from the latissimus dorsi and the group with breast reconstruction using a musculocutaneous flap from the rectus abdominis is presented in Table 3.

**Table 3.** Total scores from the Breast Evaluation Questionnaire (BEQ) among the women who underwent breast reconstruction

	%			Kruskal-Wallis test: P
	Control	LD	TRAM	
Range	14.0-88.6	40.0-91.0	22.0-87.0	0.0002
Median	50	65	61.2	
Average	48.4	62.6	59.2	

LD = reconstruction using musculocutaneous flap from the latissimus dorsi; TRAM= reconstruction using musculocutaneous flap from the rectus abdominis.

To complement the Kruskal-Wallis analysis, a multiple comparison test was conducted. This showed that the total score from the BEQ in the control-mastectomy group was 48.4%, which was significantly smaller than the scores in the breast reconstruction groups. The analysis of LD versus TRAM showed that these two groups were statistically similar. The TRAM group presented a total BEQ score of 59.2%, while the LD group presented 62.6%.^14^

## DISCUSSION

Mastectomy directly affects patients’ self-esteem, femininity and body image. Therefore, identifying these women’s degree of satisfaction and the impact of their treatment on their quality of life is of utmost interest.^2^^,^^3^

We reviewed the literature to search for specific instruments for assessing breast surgery. We found studies using seven different validated instruments that assessed the results from esthetic and reconstructive breast surgery.

One of these instruments is the Breast Evaluation Questionnaire (BEQ), which was previously validated among 1,244 women who had undergone breast augmentation.^11^ The BEQ was chosen to evaluate satisfaction with overall appearance and breast appearance for the present study because, at the time when this study was designed, it was the only validated instrument available for breast evaluation in Brazil.^12^ In addition, it offers the advantage of being self-administered, thus providing greater freedom for patients to express their perceptions and minimizing any constraints that the patients might feel regarding speaking to the surgical team, especially about scars and recurrence.

The age group from 30 to 55 years was considered because, according to the Brazilian National Cancer Institute (Instituto Nacional do Câncer, INCA), the incidence of breast cancer among women grows rapidly and progressively over this age range. Themaximum age considered was 55 years in order not to include patients in the perimenopause period, since these women present distinctive hormonal changes that possibly would interfere with the results from the study.^1^^,^^15^^,^^16^

Furthermore, demographic data such as body mass index (BMI) and age were collected to verify whether these factors might interfere in the results regarding patients’ satisfaction concerning their breasts.

Regarding age, the average for the control group was 45.4 years, whereas it was 46.7 years in the group with reconstruction using a latissimus dorsi (LD) musculocutaneous flap and 46.9 years in the transverse rectus abdominis musculocutaneous (TRAM) group. Thus, there was consistency between the three groups and the age factor was considered a low risk of interference in patients’ satisfaction regarding their breasts. These data closely match those found in the literature.^17^^,^^18^^,^^19^

The same observation can be made regarding BMI, since there was no significant difference between the groups. The average BMI was 23.9 kg/m^2^ in the control group, while in the reconstruction groups with musculocutaneous flaps from the LD and from the TRAM, the BMI was 23.3 kg/m^2^ and 23.6 kg/m^2^, respectively. Thesevalues are slightly below the average observed in the literature.^20^^,^^21^

Data about the level of education were collected, and these showed that the control group and the reconstruction groups all presented higher numbers of patients who had completed high school, while smaller numbers of them had only attended elementary school. This made it easier to apply the BEQ, since this is a self-administered instrument.^12^

Moreover, there were no significant differences between the groups with regard to marital status, skin color or occupation. Thus,these factors contributed towards greater homogeneity among the groups and lower risk of interference in the results obtained. The same was noted in the literature.^22^

Patients’ surgical results can be evaluated through their satisfaction with these results. This analysis is subjective and therefore questionnaires have been applied in an attempt to obtain an objective analysis and enable data measurement and comparison between patients.^19^^,^^23^

The BEQ was applied one year after all surgical procedures had been completed. Thus, the patients had already gone through the stage of surgical recovery and had returned to their routine. It has been shown that the results may be influenced by elation after surgery, but that six months after surgery, patients’ feelings regarding their operations (such as helplessness, isolation, fear of death, pain and mutilation) had stabilized.^21^^,^^24^^,^^25^^,^^26^

The limitation of this study was the difficulty in contacting the patients. According to the requirements of the BEQ, they need to have completed all surgical procedures before application of the questionnaire, so that the result from the survey is not affected.

The total scores from the BEQ showed that the women who had undergone breast reconstruction using LD and TRAM flaps were satisfied with their breasts. Thus, these findings were consistent with the results from previous studies evaluating satisfaction.^8^^,^^16^^,^^17^^,^^27^^,^^28^^,^^29^

According to some authors, mastectomy can cause low self-esteem and body image issues. Therefore, reconstruction is indicated for patients requiring mastectomy. These authors observed that patients’ opinions regarding their surgical results and hospital care influenced their quality of life.^24^ A scale of satisfaction applied to patients and surgeons showed that the esthetic results were better and the level of satisfaction was higher according to the patients than according to the surgeons.^28^

It was found in previous studies that reconstructions with LD and TRAM flaps resulted in symmetry with the contralateral breast. Therefore, it was concluded that both methods produced good esthetic results and improved quality of life.^2^^,^^8^^,^^17^^,^^27^ These findings corroborate the results obtained from the present study, which identified a greater level of satisfaction with reconstructed breasts, in comparison with patients without reconstruction.

Communication between doctors and patients is important because, through the guidelines given by doctors, patients can decide what kind of surgery is best for them. This increases the chances of obtaining the expected results in relation to body image and satisfaction with breasts.^30^^,^^31^

It has been seen that the numbers of indications of breast reconstruction for women who have undergone mastectomy is increasing. Therefore, there is a need for further research and interventions to ensure that patients have fair access to this important component of multidisciplinary breast cancer treatment. Reconstructions are crucial for mastectomized women, and the importance of such indications was corroborated by the results from the present study: all the patients who underwent breast tissue reconstruction, irrespective of whether this was with LD or TRAM musculocutaneous flaps, were satisfied with the results.^32^

## CONCLUSION

Within our sample, the women who underwent breast reconstruction using flaps from either the latissimus dorsi or the rectus abdominis had similar satisfaction scores.
